# Patch Test Reactions to Dental Materials: A Retrospective Single‐Centre Study

**DOI:** 10.1111/cod.70205

**Published:** 2026-06-16

**Authors:** Mojca Bizjak‐Suran, Manca Marčun, Mitja Košnik, Dejan Dinevski

**Affiliations:** ^1^ Division of Allergy University Clinic of Respiratory and Allergic Diseases Golnik Golnik Slovenia; ^2^ Faculty of Medicine, University of Ljubljana Ljubljana Slovenia; ^3^ Faculty of Medicine, University of Maribor Maribor Slovenia

**Keywords:** contact hypersensitivity, dental material allergy, dental materials, metals, methacrylates, nickel, palladium, patch testing

## Abstract

**Background:**

Patch testing is used when delayed‐type hypersensitivity to dental materials is suspected, but no European baseline series exists for patients with suspected allergy to dental materials.

**Objectives:**

To describe patch test positivity to a 24‐allergen dental series, assess the added yield of late readings at D6/7 and characterise methacrylate reactivity patterns.

**Methods:**

In this retrospective single‐centre study, 439 adults referred between 2016 and 2025 for suspected allergy to dental materials were patch tested with an institutional 24‐allergen dental series comprising 11 metals, 8 methacrylates and 5 other substances. Reactions were read on D3 and D6/7 and scored by ICDRG criteria.

**Results:**

Overall, 50.1% of patients had at least one positive reaction, and 41.0% had a positive metal reaction. Nickel (23.0%) and palladium (16.6%) were the most frequent allergens. Amongst methacrylates, HEMA (3.6%), HPMA (3.0%) and EGDMA (2.5%) were most frequent. Amongst methacrylate‐positive patients, 44.4% had two or more positive reactions, 40.7% were HEMA‐negative and 14.8% were HEMA‐negative but HPMA‐positive. Across all allergens, 19.8% of positive reactions were detectable only at D6/7, corresponding to a 24.7% relative increase in detection compared with D3 alone.

**Conclusions:**

Late readings improved detection, and methacrylate reactivity was not fully captured by HEMA alone.

## Graphical abstract

In this retrospective single‐centre study of 439 adults referred for suspected delayed‐type hypersensitivity to dental materials, 50.1% had at least one positive patch test reaction, most often to metals. Nickel (23.0%) and palladium (16.6%) pre‐dominated, whilst methacrylate positivity was uncommon (6.2%) and not fully captured by HEMA alone. A Day 6 or Day 7 reading detected an additional 19.8% of positive reactions, supporting late readings in dental patch testing.

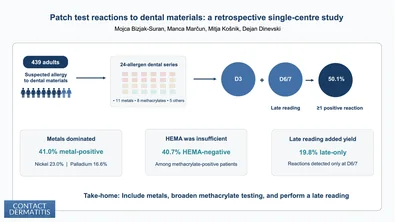

## 1 Introduction

Patch testing is used to identify contact sensitisation to dental materials. In allergic contact dermatitis, the European baseline series provides a standardised framework for routine patch testing and is updated as new evidence emerges [1]. No European baseline series exists for patients with suspected allergy to dental materials, and allergen selection still depends largely on national or local protocols and on clinical judgement regarding relevant exposures [2].

Delayed‐type hypersensitivity to dental materials may manifest as allergic contact stomatitis with presentations such as allergic gingivitis, cheilitis, burning mouth, glossodynia and oral lichenoid lesions [3, 4, 5], and remains a clinically challenging problem because these presentations may overlap with non‐allergic irritation, trauma and unrelated oral diseases.

Possible causes of delayed‐type hypersensitivity in patients with suspected dental material allergy can be grouped into three broad categories: metals, (meth)acrylates and other substances. Metals are found in restorations, crowns, bridges, cast alloys, implants, dentures and orthodontic appliances. Methacrylates are chemicals used mainly in tooth‐coloured filling materials, dental adhesives, denture‐base materials and denture repair materials. Other relevant substances may come from dental materials or oral‐hygiene products and include flavourings, fragrances and resin‐related chemicals such as carvone, eugenol, balsam of Peru, colophony and epoxy resin.

Additional data on sensitisation frequencies and co‐positivity patterns for dental allergens in patients with suspected dental material allergy would be useful for clinicians selecting a practical dental series and may help inform the future development of a European baseline series for this patient group. We describe patch test findings in patients investigated for suspected delayed‐type hypersensitivity to dental materials using a local 24‐allergen dental series. The aims were to describe allergen‐specific positivity, reaction strength, co‐positivity patterns, late‐only positivity and reaction evolution from Day 3 (D3) to Day 6 or Day 7 (D6/7).

## 2 Patients and Methods

### 2.1 Study Design and Patients

This retrospective observational study included 439 consecutive adult patients referred to our tertiary allergy clinic by dental practitioners, including general dental practitioners and specialists in maxillofacial surgery, periodontology, endodontics and prosthodontics, between October 2016 and November 2025 because of suspected delayed‐type hypersensitivity to dental materials.

### 2.2 Patch Test Allergens

Patients were patch‐tested with an institutional series of 24 allergens comprising 11 metals, 8 methacrylates and 5 other substances. Eighteen were included in the Swedish Contact Dermatitis Research Group dental screening series (marked with in Table 1) [2], and six were additional locally selected substances. Amalgam 5% pet. contained mercury together with silver, copper, tin and zinc, whereas amalgam alloying metals 20% pet. contained the same non‐mercury metals in higher proportions but excluded mercury. Amalgam was tested in 414 patients and titanium oxide in 427; all other substances were tested in all 439 patients. Full details, including sources, concentrations, vehicles and CAS numbers, are given in Table 1.

**TABLE 1:** Patch test results for 24 dental test substances.

Test substance	Conc.	CAS No.	Tested *N*	Positivity % [95% CI], *n*	Strength of pos. reactions *n* (% of pos.)			Late‐only pos. *n* (% of pos.)	Evolution *n* (% of pos.)			Doubtful *n* (% of *N*) ?+
					+	++	+++		=	↓	↑	
Metals												
Nickel sulphate^a^, ^b^	5%	10101‐97‐0	439	23.0 [19.1–27.2], *n* = 101	30 (29.7)	25 (24.8)	46 (45.5)	15 (14.9)	38 (37.6)	22 (21.8)	41 (40.6)	10 (2.3)
Palladium chloride^b^, ^c^	1%	7647‐10‐1	439	16.6 [13.3–20.4], *n* = 73	32 (43.8)	30 (41.1)	11 (15.1)	22 (30.1)	21 (28.8)	11 (15.1)	41 (56.2)	15 (3.4)
Gold sodium thiosulphate^b^, ^c^	0.5%	10210‐36‐3	439	7.3 [5.0–10.1], *n* = 32	15 (46.9)	11 (34.4)	6 (18.8)	9 (28.1)	12 (37.5)	6 (18.8)	14 (43.8)	5 (1.1)
Amalgam^c^, ^d^	5%	−	414	7.0 [4.7–9.9], *n* = 29	21 (72.4)	7 (24.1)	1 (3.4)	5 (17.2)	8 (27.6)	7 (24.1)	14 (48.3)	28 (6.8)
Potassium dichromate^b^, ^c^	0.5%	7778‐50‐9	439	6.6 [4.5–9.3], *n* = 29	22 (75.9)	6 (20.7)	1 (3.4)	9 (31.0)	6 (20.7)	8 (27.6)	15 (51.7)	12 (2.7)
Cobalt chloride^b^, ^c^	1%	7791‐13‐1	439	6.6 [4.5–9.3], *n* = 29	18 (62.1)	8 (27.6)	3 (10.3)	7 (24.1)	6 (20.7)	8 (27.6)	15 (51.7)	8 (1.8)
Ammoniated mercury^b^, ^c^	1%	10124‐48‐8	439	2.3 [1.1–4.1], *n* = 10	5 (50.0)	3 (30.0)	2 (20.0)	3 (30.0)	4 (40.0)	1 (10.0)	5 (50.0)	1 (0.2)
Amalgam alloying metals^c^, ^d^	20%	—	439	1.8 [0.8–3.6], *n* = 8	7 (87.5)	0	1 (12.5)	1 (12.5)	4 (50.0)	2 (25.0)	2 (25.0)	6 (1.4)
Ammonium heptamolybdate^c^	1%	12054‐85‐2	439	1.6 [0.6–3.3], *n* = 7	6 (85.7)	1 (14.3)	0	2 (28.6)	0	3 (42.9)	4 (57.1)	5 (1.1)
Titanium oxide^c^	0.1%	1317‐70‐0	427	1.4 [0.5–3.0], *n* = 6	6 (100)	0	0	0	1 (16.7)	4 (66.7)	1 (16.7)	8 (1.9)
Copper sulphate^c^	1% aq.	7758‐99‐8	439	0.7 [0.1–2.0], *n* = 3	2 (66.7)	1 (33.3)	0	0	0	2 (66.7)	1 (33.3)	2 (0.5)
Methacrylates												
HEMA^b^, ^c^	2%	868‐77‐9	439	3.6 [2.1–5.9], *n* = 16	9 (56.2)	5 (31.2)	2 (12.5)	5 (31.2)	5 (31.2)	5 (31.2)	6 (37.5)	2 (0.5)
HPMA^a^	2%	27813‐02‐1	439	3.0 [1.6–5.0], *n* = 13	9 (69.2)	2 (15.4)	2 (15.4)	3 (23.1)	5 (38.5)	4 (30.8)	4 (30.8)	1 (0.2)
EGDMA^b^, ^c^	2%	97‐90‐5	439	2.5 [1.3–4.4], *n* = 11	9 (81.8)	1 (9.1)	1 (9.1)	1 (9.1)	6 (54.5)	4 (36.4)	1 (9.1)	3 (0.7)
MMA^a^, ^b^	2%	80‐62‐6	439	1.8 [0.8–3.6], *n* = 8	6 (75.0)	2 (25.0)	0	1 (12.5)	5 (62.5)	1 (12.5)	2 (25.0)	4 (0.9)
BIS‐GMA^a^, ^b^	2%	1565‐94‐2	439	1.1 [0.4–2.6], *n* = 5	3 (60.0)	2 (40.0)	0	1 (20.0)	1 (20.0)	3 (60.0)	1 (20.0)	2 (0.5)
TEGDMA^b^, ^c^	2%	109‐16‐0	439	0.5 [0.1–1.6], *n* = 2	1 (50.0)	1 (50.0)	0	0	0	2 (100)	0	3 (0.7)
THFMA^a^, ^b^	2%	2455‐24‐5	439	1.1 [0.4–2.6], *n* = 5	4 (80.0)	1 (20.0)	0	1 (20.0)	0	4 (80.0)	1 (20.0)	4 (0.9)
BUDMA^b^, ^c^	2%	2082‐81‐7	439	0.5 [0.1–1.6], *n* = 2	2 (100)	0	0	0	0	2 (100)	0	4 (0.9)
Other substances												
Balsam of Peru^b^, ^c^	25%	8007‐00‐9	439	8.9 [6.4–11.9], *n* = 39	28 (71.8)	7 (17.9)	4 (10.3)	0	17 (43.6)	21 (53.8)	1 (2.6)	18 (4.1)
Carvone^a^, ^b^	5%	6485‐40‐1	439	4.8 [3.0–7.2], *n* = 21	11 (52.4)	9 (42.9)	1 (4.8)	4 (19.0)	6 (28.6)	9 (42.9)	6 (28.6)	13 (3.0)
Colophony^a^, ^b^	20%	8050‐09‐7	439	3.0 [1.6–5.0], *n* = 13	4 (30.8)	4 (30.8)	5 (38.5)	2 (15.4)	6 (46.2)	2 (15.4)	5 (38.5)	3 (0.7)
Bisphenol A epoxy resin^a^, ^b^	1%	25 068‐38‐6	439	2.5 [1.3–4.4], *n* = 11	4 (36.4)	3 (27.3)	4 (36.4)	2 (18.2)	6 (54.5)	0	5 (45.5)	3 (0.7)
Eugenol^b^, ^c^	1%	97‐53‐0	439	1.4 [0.5–3.0], *n* = 6	5 (83.3)	1 (16.7)	0	2 (33.3)	0	4 (66.7)	2 (33.3)	8 (1.8)

*Note:* Test substances are listed in descending order of positive reaction frequency. Petrolatum was used as the vehicle unless otherwise specified. Patch test results were interpreted according to ICDRG criteria: ?+ doubtful; + weak positive; ++ strong positive; +++ extreme positive. The highest reaction intensity observed between D3 and D6/7 is shown. Late‐only positivity was defined as a negative or doubtful reaction on D3 that became positive (+, ++ or +++) by D6/7. Evolution of reactions from D3 to D6/7 was categorised as plateau (=), decrescendo (↓) or crescendo (↑). Decrescendo was defined as a decrease in reaction intensity (+++, ++, +, ?+ or −), plateau as unchanged morphology (+++, ++ or +) and crescendo as an increase in reaction intensity (−, ?+, +, ++ or +++).

Abbreviations: aq., aqueous; BIS‐GMA, bisphenol A glycerolate dimethacrylate; BUDMA, 1,4‐butanediol dimethacrylate; CI, confidence interval; conc., concentration; D3, day 3; D6/7, day 6 or 7; EGDMA, ethylene glycol dimethacrylate; HEMA, 2‐hydroxyethyl methacrylate; HPMA, hydroxypropyl methacrylate; MMA, methyl methacrylate; pos., positive(s); TEGDMA, triethylene glycol dimethacrylate; THFMA, tetrahydrofurfuryl methacrylate.

^a^ Sourced from Chemotechnique (Vellinge, Sweden).

^b^ Suggested by the Swedish Contact Dermatitis Research Group [2].

^c^ Sourced from allergEAZE (SmartPractice, Tucson, Arizona).

^d^ Amalgam contained mercury (Hg) 2.5%, silver (Ag) 1.7%, copper (Cu) 0.3%, tin (Sn) 0.4% and zinc (Zn) 0.025%. Amalgam alloy metals contained silver (Ag) 13.9%, copper (Cu) 2.4%, tin (Sn) 3.5% and zinc (Zn) 0.2%.

### 2.3 Patch Test Procedure

Test substances were loaded into square allergEAZE patch test chambers at our department and applied to the upper back for 48 h. Patients removed the patches themselves after 48 h and were instructed to remark the patch borders before removal.

### 2.4 Definitions and Interpretation of Patch Test Reactions

Patch test readings were performed on D3 and D6/7 by physicians experienced in skin allergy. Reactions were scored according to International Contact Dermatitis Research Group (ICDRG) recommendations as doubtful (?+), weak positive (+), strong positive (++) or extreme positive (+++) [6]. A reaction was considered positive if it was at least + on D3 and/or D6/7. Late‐only positivity was defined as a negative or doubtful reaction on D3 and a positive reaction on D6/7. Changes in positive reactions between D3 and D6/7 were also assessed. Decrescendo was defined as a decrease in reaction intensity, plateau as unchanged morphology and crescendo as an increase in reaction intensity [7].

### 2.5 Statistical Analysis

Data were entered into an electronic database and analysed using IBM SPSS, version 25. Statistical analyses were descriptive. Descriptive statistics included frequencies, percentages and medians with interquartile ranges (IQR). Positivity rates are presented with 95% confidence intervals.

### 2.6 Ethics

The procedures were conducted in accordance with the Declaration of Helsinki. The study was approved by the National Medical Ethics Committee of the Republic of Slovenia (0120‐18/2026‐2711‐3). Patients provided written informed consent for patch testing and use of their clinical data. No identifiable personal information is included in this manuscript.

## 3 Results

### 3.1 Patient Characteristics and Overall Test Findings

Of the 439 tested patients, 85.0% were women and 88.2% were aged ≥ 40 years. The median age was 61 years (IQR 51–69). Overall, 50.1% (220/439) had at least one positive patch test reaction; 21.9% (96/439) had exactly one positive reaction and 28.2% (124/439) had two or more positive reactions.

### 3.2 Reaction Strength, Late‐Only Positivity and Reaction Evolution

Positive reactions were most often weak positive or strong positive. The highest proportions of extreme positive reactions were observed for nickel sulphate, colophony and bisphenol A epoxy resin (Table 1). Overall, 80.2% (384/479) of all positive reactions were already detectable on D3, whereas 19.8% were late‐only positive. Late‐only positivity was observed in 22.3% (73/327) of positive reactions to metals and 19.4% (12/62) of positive reactions to methacrylates. Allergen‐specific late‐only positivity and reaction evolution patterns are shown in Table 1.

### 3.3 Positive Reactions to Metals

Positive reactions to metals were common, occurring in 41.0% (180/439) of patients. Amongst these patients, 50.6% reacted to one metal and 49.4% to two or more. The most frequent metal test substances with positive reactions were nickel sulphate (23.0%), palladium chloride (16.6%), gold sodium thiosulphate (7.3%), amalgam (7.0%), potassium dichromate (6.6%) and cobalt chloride (6.6%) (Table 1). Overlap between amalgam, ammoniated mercury and amalgam alloying metals was incomplete. Amongst 29 patients with positive reactions to amalgam, 23 were negative to ammoniated mercury and 25 were negative to amalgam alloying metals. Conversely, amongst the nine patients with positive reactions to ammoniated mercury, three had no positive reaction to amalgam.

### 3.4 Methacrylate Reactions and Reactivity Profiles

Positive reactions to methacrylates were uncommon overall, occurring in 6.2% (27/439) of patients. Amongst these patients, 55.6% (15/27) had one positive methacrylate reaction and 44.4% (12/27) had two or more. HEMA was the most frequent methacrylate allergen (3.6%), followed by HPMA (3.0%) and EGDMA (2.5%); the remaining methacrylates showed positivity rates of 1.8% or less (Table 1). Individual methacrylate reactivity profiles are shown in Table 2. HEMA was negative in 40.7% (11/27) of methacrylate‐positive patients. In addition, 14.8% (4/27) were HEMA‐negative but HPMA‐positive. Several patients also showed isolated reactivity to methacrylates other than HEMA and HPMA, including MMA, EGDMA, THFMA and BUDMA.

**TABLE 2:** Individual reactivity profiles to methacrylates.

Patient no.	No. positive methacrylates (*n* = 62)	HEMA (*n* = 16)	HPMA (*n* = 13)	EGDMA (*n* = 11)	MMA (*n* = 8)	BIS‐GMA (*n* = 5)	TEGDMA (*n* = 2)	THFMA (*n* = 5)	BUDMA (*n* = 2)
1	1	+	−	−	−	−	−	−	−
2	1	−	+	−	−	−	−	−	−
3	4	+	+	+	+	−	−	−	−
4	1	+	−	−	−	−	−	−	−
5	1	−	−	−	+	−	−	−	−
6	1	−	−	−	−	−	−	+	−
7	3	−	+	+	−	+	−	−	−
8	1	+	−	−	−	−	−	−	−
9	1	++	−	−	−	−	−	−	−
10	4	+	+	+	+	−	−	−	−
11	5	++	++	+	++	+	−	−	−
12	1	−	−	−	−	−	−	−	+
13	1	−	−	+	−	−	−	−	−
14	2	−	−	−	−	−	+	+	−
15	4	++	+	+	−	+	−	−	−
16	4	+	+	+	+	−	−	−	−
17	1	+	−	−	−	−	−	−	−
18	1	−	+	−	−	−	−	−	−
19	3	+++	+++	++	−	−	−	−	−
20	3	++	+	+	−	−	−	−	−
21	5	++	++	+	++	−	−	++	−
22	1	−	+	−	−	−	−	−	−
23	2	+	−	−	−	++	−	−	−
24	1	+	−	−	−	−	−	−	−
25	8	+++	+++	+++	+	++	++	+	+
26	1	−	−	−	−	−	−	+	−
27	1	−	−	−	+	−	−	−	−

*Note:* Each row represents one patient with positive reactions to methacrylates. ‘No. positive’ indicates the number of methacrylate allergens eliciting a positive reaction in that patient. Patch test results were interpreted according to ICDRG criteria: ?+ doubtful; + weak positive; ++ strong positive; +++ extreme positive.

Abbreviations: BIS‐GMA, bisphenol A glycerolate dimethacrylate; BUDMA, 1,4‐butanediol dimethacrylate; EGDMA, ethylene glycol dimethacrylate; HEMA, 2‐hydroxyethyl methacrylate; HPMA, hydroxypropyl methacrylate; MMA, methyl methacrylate; TEGDMA, triethylene glycol dimethacrylate; THFMA, tetrahydrofurfuryl methacrylate.

### 3.5 Other‐Substance Reactions

Positive reactions to non‐metal, non‐methacrylate substances were detected most often to Balsam of Peru (8.9%), followed by carvone (4.8%), colophony (3.0%), bisphenol A epoxy resin (2.5%) and eugenol (1.4%). Reactions to these substances were generally weak or strong, although colophony and bisphenol A epoxy resin showed relatively high proportions of extreme positive reactions (Table 1).

## 4 Discussion

### 4.1 Clinical Interpretation

In this retrospective single‐centre study, half of the patients had at least one positive patch test reaction and more than one quarter had two or more positive reactions. These findings are best interpreted in the context of a selected tertiary referral population with suspected dental material allergy, rather than as prevalence estimates for an unselected dental population. Positive patch test reactions do not by themselves establish clinical relevance. In patients with oral or perioral complaints, clinical relevance is most possible when the clinical presentation is compatible with contact exposure, when the positive allergen is present in an intraoral restoration, prosthesis, orthodontic appliance, adhesive, denture material or oral‐hygiene product, and when symptoms or lesions improve after avoidance or targeted replacement of the suspected source [8, 9, 10, 11]. Because detailed information on lesion morphology, lesion topography, dental material composition, exposure to oral‐hygiene products and post‐avoidance or post‐replacement outcomes was not collected for this analysis, the present study describes sensitisation patterns rather than confirmed clinically relevant allergy.

The marked female predominance in our cohort is consistent with the higher frequency of contact sensitisation reported in women in the general population [12], and with the female predominance reported in several dental patch test cohorts, in which women accounted for 77.4%–93.3% of tested patients [8, 13, 14, 15, 16]. The high proportion of women should therefore be considered when interpreting the overall positivity rates and may limit generalisability to all patients with dental materials.

Forty‐one percent of patients had at least one positive reaction to metals. This is unsurprising given the likely extent of metal exposure from orthodontic appliances, amalgam restorations, crowns, bridgework and removable prostheses. Metal sensitisation in our cohort was generally consistent with previous dental patch test cohorts (Table 3) [8, 13, 17], whereas positivity rates in selected cohorts with suspected metal allergy or oral lichenoid disease were within a broader range (Table S1) [9, 14, 15, 16, 18, 19]. For metals such as nickel, cobalt and chromium, clinical relevance is most likely when positive patch test reactions correspond to metals present in the patient's dental materials and to mucosal or perioral lesions in close anatomical relation to those materials [9, 20]. Non‐allergic causes should also be considered, as mucosal inflammation in patients with cobalt‐chromium prostheses has been attributed mainly to candidiasis, poor oral hygiene and ill‐fitting prostheses rather than allergy [21].

**TABLE 3:** Patch test positivity in the current study compared with previous dental patch test cohorts.

Test substance	Current study % (*n/N*), conc.	Forkel [13] % (*n/N*), conc.	Al‐Gawahiri [8] % (*n/N*), conc.	Kanerva [17] % (*n/N*), conc.
	Suspected reactions to dental materials	Allergic stomatitis	Intra‐/perioral complaints	Suspected reactions to dental materials
Metals				
Nickel sulphate	23.0 (101/439), 5%	28.6 (124/433), 5%	23.3 (84/360), 5%	14.6 (248/1693), NR
Palladium chloride	16.6 (73/439), 1%	21.4 (94/440), 1%	13.9 (50/360), 1% aq.	4.2 (107/2540), NR
Gold sodium thiosulphate	7.3 (32/439), 0.5%	8.9 (39/440), 0.25%	—	7.7 (345/4508), NR
Potassium dicyanoaurate^a^	—	—	9.2 (33/360), 100 ppm aq.	—
Amalgam	7.0 (29/414), 5%	10.9 (48/439), 5%	—	1.1 (4/367), NR
Potassium dichromate	6.6 (29/439), 0.5%	4.8 (21/441), 0.5%	3.6 (13/360), 0.5%	—
Cobalt chloride	6.6 (29/439), 1%	8.2 (36/438), 1%	10.0 (36/360), 1%	4.1 (69/1663), NR
Ammoniated mercury	2.3 (10/439), 1%	6.3 (28/442), 1%	2.2 (8/360), 1%	13.0 (100/767), NR
Mercury	—	—	6.7 (24/360), 0.5%	10.3 (259/2519), NR
Mercuric/mercury chloride	—	—	—	8.9 (139/1568), NR
Amalgam alloying metals	1.8 (8/439), 20%	0.2 (1/441), 20%	—	0.0 (0/73), NR
Ammonium molybdate/heptamolybdate	1.6 (7/439), 1%	—	1.4 (5/360), 1% aq.	—
Titanium oxide	1.4 (6/427), 0.1%	—	—	—
Copper sulphate	0.7 (3/439), 1% aq.	0.5 (2/437), 1% aq.	—	0.2 (6/2611), NR
Methacrylates				
HEMA	3.6 (16/439), 2%	4.8 (21/442), 1%	4.2 (15/360), 2%	2.8 (69/2446), NR
HPMA	3.0 (13/439), 2%	4.3 (19/437), 2%	—	—
EGDMA	2.5 (11/439), 2%	4.1 (18/441), 2%	3.1 (11/360), 2%	1.9 (46/2444), NR
MMA	1.8 (8/439), 2%	4.1 (18/442), 2%	2.8 (10/360), 2%	1.1 (28/2607), NR
BIS‐GMA	1.1 (5/439), 2%	—	1.1 (4/360), 2%	0.2 (5/2407), NR
TEGDMA	0.5 (2/439), 2%	0.7 (3/442), 2%	2.5 (9/360), 2%	0.5 (12/2586), NR
THFMA	1.1 (5/439), 2%	0.7 (3/438), 2%	1.9 (7/360), 2%	0.7 (17/2406), NR
BUDMA	0.5 (2/439), 2%	0.9 (4/439), 2%	1.7 (6/360), 2%	0.5 (13/2408), NR
Other substances				
Balsam of Peru	8.9 (39/439), 25%	11.4 (50/439), 25%	8.3 (30/360), 25%	—
Carvone	4.8 (21/439), 5%	—	—	—
Colophony	3.0 (13/439), 20%	4.3 (19/441), 20%	3.3 (12/360), 20%	1.5 (30/1981), NR
Epoxy resin	2.5 (11/439), 1%	1.4 (6/441), 1%	1.7 (6/360), 1%	0.1 (1/1356), NR
Eugenol	1.4 (6/439), 1%	—	0.3 (1/360), 2%	0.4 (10/2527), NR

*Note:* Numerical values are presented as positivity (%; *n*/*N*) and test concentration. Petrolatum was used as the vehicle unless otherwise specified. The symbol — indicates that the substance was not tested or that no corresponding data were available in the source study. NR indicates that the test concentration was not reported. Patient populations are specified in the second header row.

Abbreviations: aq., aqueous; BIS‐GMA, bisphenol A glycerolate dimethacrylate; BUDMA, 1,4‐butanediol dimethacrylate; EGDMA, ethylene glycol dimethacrylate; HEMA, 2‐hydroxyethyl methacrylate; HPMA, hydroxypropyl methacrylate; MMA, methyl methacrylate; NR, not reported; TEGDMA, triethylene glycol dimethacrylate; THFMA, tetrahydrofurfuryl methacrylate.

^a^ Synonym: potassium gold cyanide.

Palladium was the second most frequent allergen overall (16.6%) (Table 1) and was also amongst the most frequent allergens in other dental patch test cohorts (13.9%–21.4%) (Table 3) [8, 13]. Because palladium is widely used in dental casting alloys, dental exposure is likely to be important [22, 23]. However, concomitant nickel and palladium reactivity is frequent [22], and cross‐reactivity has been supported by oral nickel challenge studies showing flare‐up reactions at previous palladium patch test sites [24]. Palladium sensitisation may also be underdetected when patch testing relies only on palladium dichloride [22], as in our study. A positive palladium reaction should be interpreted together with nickel reactivity, the type of palladium test salt used and evidence of palladium‐containing dental alloys in the mouth.

Gold sodium thiosulphate showed a positivity rate close to those reported in other dental patch test cohorts (Table 3) [13, 17], but lower than in selected oral disease cohorts (Table S1) [18, 19]. Gold sensitisation has been considered clinically relevant in selected patients with stomatitis and oral lichenoid lesions, particularly when lesions improve after replacement of gold‐containing restorations [25]. Patch test reactions to gold sodium thiosulphate may appear late and persist for weeks, so early readings may miss relevant reactions [26, 27]. In the current study, 28.1% of positive reactions to gold sodium thiosulphate were late‐only positive. Gold sodium thiosulphate positivity may be clinically meaningful in selected patients, particularly when compatible oral lesions occur near gold‐containing restorations; however, the result should be interpreted cautiously because the test reagent may not fully represent metallic gold present intraorally [28].

Amalgam‐related findings may remain relevant despite the declining use of amalgam due to EU mercury legislation, as many patients still have older restorations [8]. In our cohort, positivity rates were 7.0% for amalgam, 2.3% for ammoniated mercury and 1.8% for amalgam alloying metals, falling within or close to the ranges reported in other dental patch test cohorts (Table 3) [8, 13, 17]. Higher amalgam‐ and mercury‐related positivity has been described in oral lichenoid disease, particularly when lesions are in close topographic contact with restorations [10, 19], and improvement after removal supports clinical relevance in selected cases [10, 11]. Overall, these findings support a cautious interpretation: amalgam‐related oral disease may reflect true delayed hypersensitivity in selected patients, but local irritation and other non‐allergic mechanisms may also contribute [10, 11, 19]. Because overlap between amalgam, ammoniated mercury, and amalgam alloying metals was incomplete, ammoniated mercury alone would probably miss some amalgam‐related sensitisations. Additionally, ammoniated mercury is an imperfect marker for sensitisation to metallic mercury in dental amalgam, and positive reactions may also reflect sensitisation to mercury‐containing antiseptics or topical preparations, cross‐reactivity with amalgam‐related exposure or previous non‐dental mercury exposure. Metallic mercury itself was not chosen for the dental series used in our clinic during the study period.

Titanium was an infrequent sensitiser, with titanium oxide 0.1% positive in 1.4% of tested patients. Previous studies likewise suggest that titanium hypersensitivity is uncommon relative to other dental metals, although reported frequencies vary across selected dental implant and broader populations investigated for suspected dental material allergy [15, 29, 30]. Interpretation remains difficult because different studies have used different titanium test preparations and because the clinical relevance of isolated positive results is often uncertain [15, 30]. Therefore, titanium allergy appears possible but uncommon, and positive results should be interpreted with caution in relation to implant history, symptoms, timing and alternative explanations.

Methacrylate sensitisation was uncommon overall, with 6.2% of patients showing at least one positive methacrylate reaction. Our study showed that structurally related methacrylates cannot be assumed to function as interchangeable screening markers. HEMA was the most frequent methacrylate allergen, consistent with previous studies of patients investigated for suspected dental material allergy [8, 31], but captured only 59.3% of patients with positive reactions to methacrylates. This contrasts with the Swedish series of Goon et al., in which 2‐HEMA identified 96.7% of patients with positive reactions to methacrylates and the addition of BIS‐GMA increased the yield to 100% [31]. In our cohort, HPMA identified additional patients with positive reactions to methacrylates, including 14.8% who were HEMA‐negative but HPMA‐positive. In the allergic stomatitis cohort reported by Forkel et al., HEMA, HPMA, EGDMA and MMA were amongst the most relevant methacrylates [13]. Clinically, these findings support testing a broader methacrylate panel when exposure to composite resins, adhesives, denture‐base materials, splints or repair materials is suspected.

Non‐metal, non‐methacrylate substances also deserve attention. Balsam of Peru, carvone, colophony, bisphenol A epoxy resin and eugenol all elicited positive reactions, with Balsam of Peru amongst the more frequent positive test substances overall. These findings are consistent with previous studies supporting inclusion of natural substances such as propolis and Balsam of Peru [13], reports of oral contact allergy to carvone‐containing dental consumer products [13], and epoxy‐related dental resin systems [32]. Tin was not included as a separate test substance in our institutional series, which is a limitation because stannous fluoride and other tin‐containing oral‐hygiene products have been reported as causes of cheilitis and contact allergy [33, 34]. These findings support considering oral‐hygiene products, flavourings, fragrances, resin‐related substances and, when clinically indicated, tin‐containing products in addition to restorative materials.

Late‐only positivity was not uncommon, indicating that reliance on an early reading alone would have missed a relevant proportion of reactions. Previous studies likewise suggest that late readings are useful for HEMA [31], palladium [19], gold [25, 27] and mercury compounds [19]. Although some allergen‐specific proportions in our cohort were based on small numbers, the overall pattern supports inclusion of a late reading in dental patch testing, particularly when clinical suspicion remains high.

### 4.2 Strengths and Limitations

The strengths of the present study include the relatively large cohort for a single‐centre dental patch test study, the use of a structured 24‐allergen dental series covering metals, methacrylates and other relevant substances, and the inclusion of both D3 and D6/7 readings, which allowed assessment of late‐only positivity and reaction evolution. The methacrylate‐focused analyses, particularly the individual reactivity profiles, also provide detail that is often missing from broader descriptive dental patch test studies. The main limitation is that clinical relevance could not be assessed systematically, because detailed data on lesion morphology, lesion topography, dental material composition, exposure to oral‐hygiene products and post‐avoidance or post‐replacement outcomes were not collected for this analysis. In addition, the tested substances may not fully represent the chemical form, concentration or bioavailability of materials actually present in the oral cavity and some causative allergens may not have been included in the series. These limitations mean that both clinically irrelevant sensitisation and false‐negative or only partially informative results are possible. Because patients removed the patches themselves after 48 h, very early decrescendo reactions may also have been missed.

## 5 Conclusions

Overall, these findings support dental patch testing that includes metals, a broader methacrylate panel than HEMA alone, selected non‐metal substances and a late reading. Nickel and palladium were the most prevalent allergens and HPMA and EGDMA increased methacrylate detection beyond HEMA. Interpretation should be integrated with lesion morphology, lesion topography, exposure history and precise information from the treating dental clinician about the composition of intraoral materials.

## Author Contributions

**Mojca Bizjak‐Suran:** conceptualization, data curation, formal analysis, investigation, methodology, project administration, resources, supervision, visualization, writing – original draft, writing – review and editing. **Mitja Košnik:** investigation, resources, writing – review and editing. **Manca Marčun:** data curation, writing – review and editing. **Dejan Dinevski:** formal analysis, visualization, writing – review and editing.

## Funding

This research was supported by the Slovenian Research and Innovation Agency (P3‐0360).

## Ethics Statement

The study was approved by the National Medical Ethics Committee of the Republic of Slovenia (0120‐18/2026‐2711‐3).

## Consent

Patients provided written informed consent for patch testing and use of their clinical data. No identifiable personal information is included in this manuscript.

## Conflicts of Interest

Mojca Bizjak‐Suran is or recently was a speaker and adviser for Novartis and Swixx Biopharma, outside the submitted work. The other authors declare no conflicts of interest.

## Supporting information


**Table S1:** Metal patch test positivity in selected metal‐focused or oral disease cohorts compared with the current study. The table summarises source‐study details, patient populations, positivity rates and patch test concentrations.

## Data Availability

The data that support the findings of this study are available on request from the corresponding author. The data are not publicly available due to privacy or ethical restrictions.
