# Supplementary material for: Patch Test Reactions to Dental Materials: A Retrospective Single‐Centre Study
Source: Contact Dermatitis. 2026 Jun 16;95(4):386–95. doi: 10.1111/cod.70205 (PMC13551403; doi:10.1111/cod.70205)
Supplement: Supplementary file 1 [file COD-95-386-s001.docx]

**Table S1.** Metal patch test positivity in selected metal-focused or oral disease cohorts compared with the current study.

| **Test substance** | **Current study**  **% (*n/N*), conc.** | **Itoh 2020^14^**  **% (*n/N*), conc.** | **Hosoki 2018^15^**  **% (*n/N*), conc.** | **Raap 2009^9^**  **% (*n/N*), conc.** | **Kim 2015^18^**  **% (*n/N*), conc.** | **Tsushima 2022^16^**  **% (*n/N*), conc.** | **Thanyavuthi 2016^19^**  **% (*n/N*), conc.** |
| --- | --- | --- | --- | --- | --- | --- | --- |
|  | Suspected dental material allergy | Pretreatment metal allergy concern | Suspected dental  metal allergy | Suspected dental metal allergy | Mixed oral disease | OLL | OLL |
| Nickel sulphate | 23.0 (101/439), 5% | 29.0 (17/59), 5% | 31.6 (NR/270), 5% | 4.9 (10/206), 5% | 25.0 (11/44), NR | 40.0 (12/30), NR | 28.3 (15/53), 5% |
| Palladium chloride | 16.6 (73/439), 1% | 10.0 (6/59), 1% | 34.2 (NR/270), 1% | 4.9 (10/206), 1% | 6.8 (3/44), NR | 56.7 (17/30), NR | 9.4 (5/53), 2% |
| Gold compounds^†^ | 7.3 (32/439), 0.5% | 3.4 (2/59), 0.2% | 8.6 (NR/270), 0.2% | 4.9 (10/206), 0.25% | 25.0 (11/44), NR | 33.3 (10/30), NR | 28.3 (15/53), 2% |
| Amalgam | 7.0 (29/414), 5% | – | – | 1.9 (4/206), 5% | – | – | – |
| Potassium dichromate | 6.6 (29/439), 0.5% | 5.0 (3/59), 0.5% | 33.1 (NR/270), 0.5% | – | 22.7 (10/44), NR | 16.7 (5/30), NR | 11.3 (6/53), 0.5% |
| Cobalt chloride | 6.6 (29/439), 1% | 17.0 (10/59), 2% | 23.0 (NR/270), 2% | 1.9 (4/206), 1% | 15.9 (7/44), NR | 30.0 (9/30), NR | 5.7 (3/53), 1% |
| Mercury compounds^‡^ | 2.3 (10/439), 1% | 1.7 (1/59), 0.05% | 10.0 (NR/270), 0.05% | 1.9 (4/206), 1% | 4.5 (2/44), NR | – | 35.8 (19/53), 0.5% |
| Amalgam alloying metals | 1.8 (8/439), 20% | – | – | 0.5 (1/206), 20% | – | – | – |
| Molybdenum compounds^§^ | 1.6 (7/439), 1% | – | 15.6 (NR/270), 1% | – | – | – | – |
| Titanium oxide | 1.4 (6/427), 0.1% | 0.0 (0/59), 0.1% | 0.4 (NR/270), 30% | – | – | 3.3 (1/30), NR | – |
| Copper sulphate | 0.7 (3/439), 1% | 2.0 (1/59), 1% | 4.5 (NR/270), 1% | – | 4.5 (2/44), NR | – | 32.0 (17/53), 2% |

*Note*: Patient populations are specified in the second header row. Numerical values are presented as positivity (%; n/N) and test concentration. The symbol – indicates that the substance was not tested or that no corresponding data were available in the source study. NR indicates that the numerator and/or denominator or the test concentration was not reported.

^†^ Gold compounds comprised different gold test substances across studies, including gold sodium thiosulphate, tetrachloroauric/chloroauric acid, gold chloride, and potassium dicyanoaurate. In the current study, gold was represented by gold sodium thiosulphate.

^‡^ Mercury compounds comprised different mercury test substances across studies, including ammoniated mercury, mercury, metallic mercury, and mercuric/mercury chloride. In the current study, mercury was represented by ammoniated mercury.

^§^ Molybdenum compounds comprised different molybdenum test substances across studies, including ammonium molybdate/heptamolybdate and molybdenum chloride. In the current study, molybdenum was represented by ammonium heptamolybdate.

*Abbreviations*: NR, not reported; OLL, oral lichenoid lesions.

**References:**

9.	Raap U, Stiesch M, Reh H, Kapp A, Werfel T. Investigation of contact allergy to dental metals in 206 patients. Contact Dermatitis. 2009;60(6):339-343.

14.	Itoh E, Furumura M, Furue M. Rate of actual metal allergy prior to dental treatment in subjects complaining of possible metal allergy. Asian Pac J Allergy Immunol. 2020;38(3):186-189.

15.	Hosoki M, Nishigawa K, Tajima T, Ueda M, Matsuka Y. Cross-sectional observational study exploring clinical risk of titanium allergy caused by dental implants. J Prosthodont Res. 2018;62(4):426-431.

16.	Tsushima F, Sakurai J, Shimizu R, Kayamori K, Harada H. Oral lichenoid contact lesions related to dental metal allergy may resolve after allergen removal. J Dent Sci. 2022;17(3):1300-1306.

18.	Kim TW, Kim WI, Mun JH, et al. Patch Testing with Dental Screening Series in Oral Disease. Ann Dermatol. 2015;27(4):389-393.

19.	Thanyavuthi A, Boonchai W, Kasemsarn P. Amalgam Contact Allergy in Oral Lichenoid Lesions. Dermatitis. 2016;27(4):215-221.
